# A meta-analysis on efficacy and safety: single-balloon *vs.* double-balloon enteroscopy

**DOI:** 10.1093/gastro/gov003

**Published:** 2015-02-18

**Authors:** Vaibhav Wadhwa, Saurabh Sethi, Sumeet Tewani, Sushil Kumar Garg, Douglas K. Pleskow, Ram Chuttani, Tyler M. Berzin, Nidhi Sethi, Mandeep S. Sawhney

**Affiliations:** ^1^Department of Internal Medicine, Fairview Hospital, Cleveland Clinic, Cleveland, OH, USA, ^2^Division of Gastroenterology, Beth Israel Deaconess Medical Center and Harvard Medical School, Boston, MA, USA and ^3^Division of Basic and Translational Research, Department of Surgery, University of Minnesota, Minneapolis, MN, USA

**Keywords:** small bowel enteroscopy, single-balloon enteroscopy, double-balloon enteroscopy, meta-analysis, outcomes

## Abstract

**Background and aim:** Double-balloon enteroscopy (DBE) and single-balloon enteroscopy (SBE) are new techniques capable of providing deep enteroscopy. Results of individual studies comparing these techniques have not been able to identify a superior strategy. Our aim was to systematically pool all available studies to compare the efficacy and safety of DBE with SBE for evaluation of the small bowel.

**Methods:** Databases were searched, including PubMed, Embase, and the Cochrane Central Register of Controlled Trials. The main outcome measures were complete small-bowel visualization, diagnostic yield, therapeutic yield, and complication rate. Statistical analysis was performed using Review Manager (RevMan version 5.2). Meta-analysis was performed using fixed-effect or random-effect methods, depending on the absence or presence of significant heterogeneity. We used the χ^2^ and I^2^ test to assess heterogeneity between trials. Results were expressed as risk ratios (RR) or mean differences with 95% confidence intervals (CI).

**Results:** Four prospective, randomized, controlled trials with a total of 375 patients were identified. DBE was superior to SBE for visualization of the entire small bowel [pooled RR = 0.37 (95% CI: 0.19–0.73; *P = *0.004)]. DBE and SBE were similar in ability to provide diagnosis [pooled RR = 0.95 (95% CI: 0.77–1.17; *P = *0.62)]. There was no significant difference between DBE and SBE in therapeutic yield [pooled RR = 0.78 (95% CI: 0.59–1.04; *P = *0.09)] and complication rate [pooled RR = 1.08 (95% CI: 0.28–4.22); *P = *0.91].

**Conclusions:** DBE was superior to SBE with regard to complete small bowel visualization. DBE was similar to SBE with regard to diagnostic yield, ability to provide treatment and complication rate, but these results should be interpreted with caution as they is based on very few studies and the overall quality of the evidence was rated as low to moderate, due to the small sample size.

## Introduction

Until recently, the small bowel was considered to be inaccessible using conventional endoscopy techniques. In 2001, Yamamoto *et al.* introduced the double-balloon enteroscopy (DBE) system to examine the small bowel [1]. DBE provides deep enteroscopy by employing a flexible overtube and two balloons, one on the tip of the endoscope and one on the overtube. The technique of alternating push-and-pull maneuvers allows the small bowel to thread on to the overtube, and the endoscope can accomplish both diagnostic and therapeutic functions during the same procedure [2–4]; however, DBE is found to have some technical issues, including complex, cumbersome preparation and handling. In 2008, a novel, simplified system of single-balloon enteroscopy (SBE) was introduced, using one instead of two balloons [5, 6]. Instead of the endoscope tip balloon in DBE, SBE involves angling the endoscope tip or power suction to achieve stable positioning in the small bowel. It has been suggested that SBE requires less preparation and examination time; however, there are concerns that it may also be less efficient than DBE for deep intubation of the small bowel. To date, several studies have compared the performance of these two techniques; however, the results of individual comparative studies have not identified a dominant strategy. We propose that systematically pooling all available studies may provide a better understanding of the performance of these procedures. The objective of our study was to perform a systematic review and meta-analysis to assess the efficacy and safety of DBE and SBE for evaluation of the small bowel.

## Materials and methods

### Search strategy

A systematic literature search of the MEDLINE (1966 through June 31^st^, 2014), Embase (1988 through June 31^st^, 2014), and Web of Science (1993 through June 31st, 2014) databases and the Cochrane Central Register of Controlled Trials, updated to June 31^st^ 2014, was conducted. Keywords—including small bowel enteroscopy, deep enteroscopy, single-balloon enteroscopy and double-balloon enteroscopy—were used to identify the relevant articles. We also manually searched the abstracts from major gastroenterology conferences, i.e. the Digestive Disease Week and the American College of Gastroenterology conference (2003–2013).

### Selection of Studies

One author, Vaibhav Wadhwa (VW) inspected all abstracts of studies identified as above, to determine potentially relevant reports. In addition—and to ensure reliability—Mandeep S. Sawhney (MSS) inspected 100% all of the identified abstracts. Where disagreement existed as to the potential relevance of a particular report, we resolved this through discussion. Where doubt persisted, we retrieved the full text of the report for examination. We retrieved the full text of all those reports judged to be potentially relevant for further assessment, after which VW and MSS in turn inspected them and independently decided whether or not they met the inclusion criteria.

### PRISMA flow diagram

We included a flow diagram to illustrate the results of searches of each databases, conference proceedings, the process of screening and selecting studies for inclusion in the review in agreement with the Preferred Reporting Items for Systematic Reviews and Meta-Analyses (PRISMA) guidelines for search and reporting processes. Our search included articles published since 1990, to include contemporary information, and was open to all research designs in order for the search to be comprehensive.

### Inclusion and exclusion criteria

Only randomized, controlled trials (RCTs) in adult patients aged 18 years or older who underwent small bowel enteroscopy, published as full articles or meeting abstracts in peer-reviewed journals, were considered. The selection criteria were (i) studies that examined the efficacy and safety of SBE and DBE, and (ii) data not duplicated in another manuscript. Inclusion was not otherwise restricted by study size or language. All studies that compared either technique with capsule endoscopy, push enteroscopy, spiral enteroscopy, or other technology were excluded.

### Data extraction

Data were independently abstracted on to a specifically designed form by two reviewers, VW and MSS. The following data were collected from each study: study design, year of publication, country of the population studied, primary outcome reported, type of small bowel enteroscopy, total number of persons in each group (SBE *vs.* DBE), and single-center or multi-center trial. Agreement between investigators was excellent (kappa = 0.93). When incomplete information was available, attempts were made to contact the corresponding authors of the studies for additional information.

### Outcomes assessed

The outcome measures were completed small bowel visualization, diagnostic yield, therapeutic yield, and complication rate.

### Assessment of risk of bias in included studies

To understand the risk of bias in individual studies, a formal quality assessment of studies was performed. The methodological quality of the RCTs was independently assessed by two authors (SS and MSS) using the scale validated by Jadad *et al.* [7] and scored from 0 to 5, *viz.* randomization (0–2 points), blinding (0–2 points), and full accounting of all patients (0–1 point); a higher score indicated better quality.

Two authors also independently assessed the methodological quality of each included study, using the Cochrane Collaboration’s ‘Risk of Bias’ tool [8]. The study features we assessed were the following: random sequence generation, allocation concealment, blinding of participants and investigators, blinding of outcome assessment, incomplete outcome data, selective reporting and ‘other bias’.

We recorded each of these factors as ‘low risk’, ‘high risk’ or ‘unclear risk’, with a brief overview provided in table format. If ‘unclear’, we attempted to seek clarification from the trial authors. After this process, we gave each paper an overall quality assessment grade of low, high or unclear risk of bias. We used the principles of the grading of recommendations assessment, development and evaluation (GRADE) system to assess the quality of the body of evidence associated with specific outcomes and constructed a Summary of Findings (SoF) table.

### Statistical analysis

Statistical analysis was performed using Review Manager (RevMan version 5.2), the Cochrane Collaboration’s software for preparing and maintaining Cochrane systematic reviews. Meta-analysis was performed using fixed-effect or random-effect methods, depending on the absence or presence of significant heterogeneity. We used the χ^2^ and I^2^ test to assess heterogeneity between trials. A *P* < 0.05 or I^2^ > 50% was defined as significant heterogeneity. Potential publication bias was examined by funnel plot. Results were expressed as risk ratios (RR) or mean differences (MD) with 95% confidence intervals (CI). A *P* < 0.05 was considered statistically significant.

## Results

### Literature search

**Figure 1.**
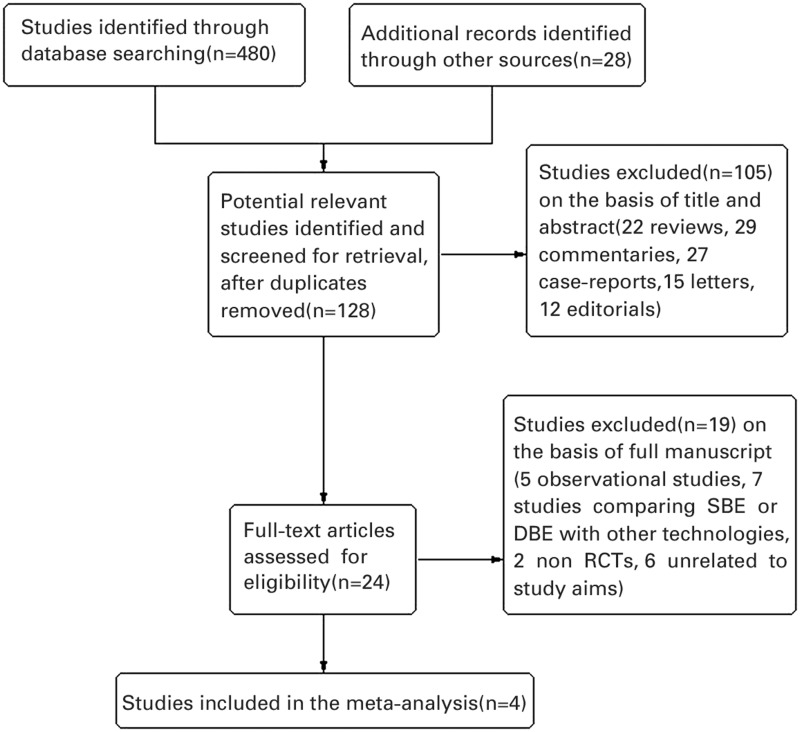
Flow diagram of included and excluded trials.

Figure 1 shows the process of study selection. Of the 128 individual studies identified using our search criteria, four fulfilled our inclusion criteria and were included in the meta-analysis. Finally, four randomized, controlled trials, covering a total of 387 patients (186 having had SBE, 201 having had DBE) fulfilled our inclusion criteria [9–12]. Of these, one trial was from Japan [12], one from Australia [10], and two were from Germany [9, 11]. All eligible articles were reported in the form of full-text articles.

### Study characteristics

The characteristics of the four included studies are summarized in Table 1. The indications for SBE and DBE in these trials were suspected small bowel diseases. All were randomized, controlled trials. Quality assessment revealed two included studies with a Jadad score of 4, while the other two scored 3. All four trials therefore had a Jadad score of 3 or more, indicating good study design or high quality of report. The differences in small bowel findings between SBE and DBE in each study are shown in Table 2.

**Table 1.** Characteristics of studies included in the meta-analysis

RCTs	Year/country	Sample size		Insertion depth (cm)*		Complete enteroscopy rate		Diagnostic yield		Therapeutic yield		Complication rate		Jadad score
		SBE	DBE	SBE	DBE	SBE	DBE	SBE	DBE	SBE	DBE	SBE	DBE	
Takano *et al.*	2011/Japan	18	20	°	°	0/14 (0%)	8/14 (57%)	11/18 (61%)	10/20 (50%)	5/18 (28%)	7/20 (35%)	1/18 (5.6%)	1/20 (5%)	3
Domagk *et al.*	2011/Germany	65	65	373 (100–620)	360 (180–550)	7/65 (11%)	12/65 (18%)	24/65 (37%)	28/65 (43%)	3/65 (5%)	6/65 (9%)	0/65 (0%)	0/65 (0%)	4
May *et al.*	2010/Germany	50	50	°	°	11/50 (22%)	33/50 (66%)	21/50 (42%)	26/50 (52%)	24/50 (48%)	36/50 (72%)	2/50 (4%)	2/50 (4%)	3
Efthymiou *et al.*	2012/Australia	53	66	203.8 ± 87.6	234.1 ± 99.3	°	°	30/53 (57%)	35/66 (53%)	16/53 (33%)	17/66 (26%)	1/53 (1.9%)	1/66 (1.5%)	4

DBE = double-balloon enteroscopy; RCTs = randomized controlled trials; SBE = single-balloon enteroscopy.

*Values presented as mean ± standard deviation or median (95% confidence interval).

**Table 2.** Comparison of small bowel findings in the four trials

RCTs	Vascular malformations		Erosions/ulcerations		Diverticula		Polyps		Malignancy		Other		Normal	
	SBE	DBE	SBE	DBE	SBE	DBE	SBE	DBE	SBE	DBE	SBE	DBE	SBE	DBE
Takano *et al.*	3	3	5	5	1	0	1	2	0	0	1	0	7	10
Domagk *et al.*	3	7	4	7	0	0	3	3	0	0	14	11	°	°
May *et al.*	9	7	8	9	2	5	1	2	1	3	°	°	°	°
Efthymiou *et al.*	14	13	5	8	°	°	6	9	°	°	2	1	22	31

DBE = double-balloon enteroscopy; RCTs = randomized controlled trials; SBE = single-balloon enteroscopy.

### Completed enteroscopy rate

**Figure 2.**
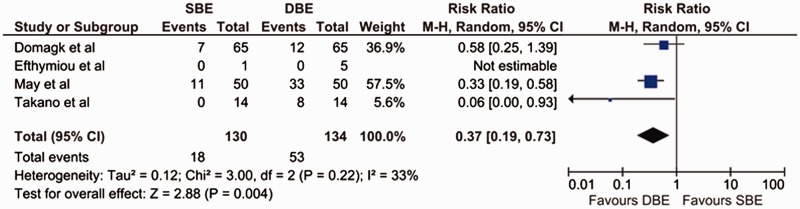
Forest plot of meta-analysis, comparing SBE and DBE for rate of completed enteroscopy. DBE = double-balloon enteroscopy; SBE = single-balloon enteroscopy.

The rate of completed small bowel enteroscopy was measured in all four studies. The random effects meta-analysis of the rate of completed small bowel enteroscopy had a heterogeneity χ^2^ value of 3.0 (*P = *0.22) and I^2^ of 33%, demonstrating evidence of statistical heterogeneity within this group of clinical trials. We found that DBE was superior to SBE in terms of its the ability to visualize the entire small bowel [pooled RR = 0.37 (95% CI: 0.19–0.73; *P = *0.004)] (Figure 2).

### Diagnostic yield

**Figure 3.**
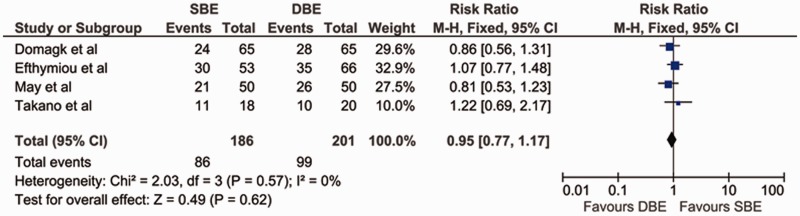
Forest plot of meta-analysis comparing SBE and DBE for diagnostic yield. DBE = double-balloon enteroscopy; SBE = single-balloon enteroscopy.

The diagnostic yield was measured in all four studies. The details of the findings are included in Table 2. The findings of Domagk *et al.* [9] & May *et al.* [11] favored DBE, while Efthymiou *et al.* [10] & Takano *et al.* [12] favored SBE; however the results were not statistically significant in any study. Our meta-analysis also revealed that DBE and SBE were similar with regard to ability to provide diagnosis [pooled RR = 0.95 (95% CI: 0.77–1.17; *P = *0.62)]. The χ^2^ was 2.03 (*P = *0.57) and I^2^ was 0%, which indicated no significant heterogeneity between the studies with regard to diagnostic yield (Figure 3).

### Therapeutic yield

**Figure 4.**
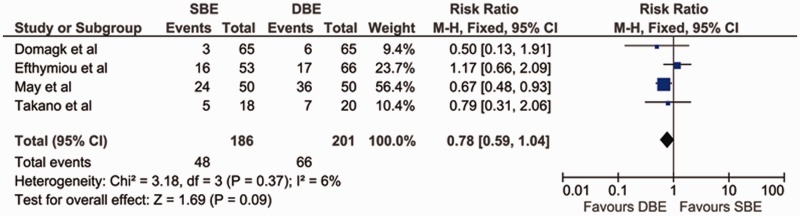
Forest plot of meta-analysis comparing SBE and DBE for therapeutic yield. DBE = double-balloon enteroscopy; SBE = single-balloon enteroscopy.

The therapeutic yield was measured in all four studies. The findings of three favored DBE; the difference was statistically significant in the study by May *et al.* [11] but not in the trials by Domagk *et al.* [9] and Takano *et al.* [12]. The findings by Efthymiou *et al.* favored SBE but they were not statistically significant [10]. On pooling the data, there was no significant difference between DBE and SBE in their ability to provide endoscopic therapy [pooled RR = 0.78 (95% CI: 0.59–1.04; *P = *0.09)]. The χ^2^ was 3.18 (*P = *0.37) and I^2^ was 6%, which indicated no significant heterogeneity between the studies with regard to therapeutic yield (Figure 4).

### Complication rate

**Figure 5.**
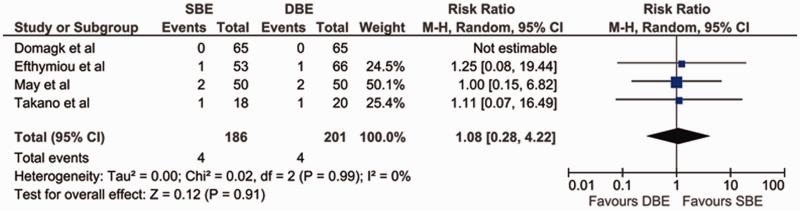
Forest plot of meta-analysis comparing SBE and DBE for complication rate. DBE = double-balloon enteroscopy; SBE = single-balloon enteroscopy.

The complication rate was measured in all four studies, and none reported severe adverse events, such as perforation, bleeding or pancreatitis. In the May *et al.* trial, three patients (one in the DBE group and two in the SBE group) developed severe abdominal pain following the procedure, and one patient in the DBE group had a brief transient intra-procedure desaturation that did not require intubation or cessation of the procedure [11]. In the trial by Takano *et al.,* one patient in the DBE group developed Mallory-Weiss syndrome, and one patient in the SBE group developed hyperamylasemia [12]. In the trial by Efthymiou *et al.,* there were two cases of post-procedural abdominal pain, one with SBE and one with DBE. In both cases, the pain was self-limiting and probably secondary to air retention [10]. The differences in complication rates were not significant in any of the studies. The meta-analysis also demonstrated that there was no significant difference between DBE and SBE with regard to complication rate [pooled RR = 1.08 (95% CI: 0.28–4.22; *P = *0.91)]. The χ^2^ was 0.02 (*P = *0.99) and I^2^ was 0%, which indicated no significant heterogeneity between the studies with regard to complication rate (Figure 5).

### Set-up time

The set-up time needed for preparation of the enteroscope for SBE and DBE was evaluated in two of the four studies. In both, it was shown that the set-up time using the single-balloon technique was significantly shorter than with the double-balloon [3 min (3–5 min) for SBE *vs.* 11 min (11–13 min) for DBE; 6 ± 2 min for SBE *vs.* 10 ± 3 min for DBE] [10, 11]. Since Efthymiou *et al.* did not report standard deviation for set-up time [10], pooled analysis of these two studies was not possible.

### Publication bias

**Figure 6.**
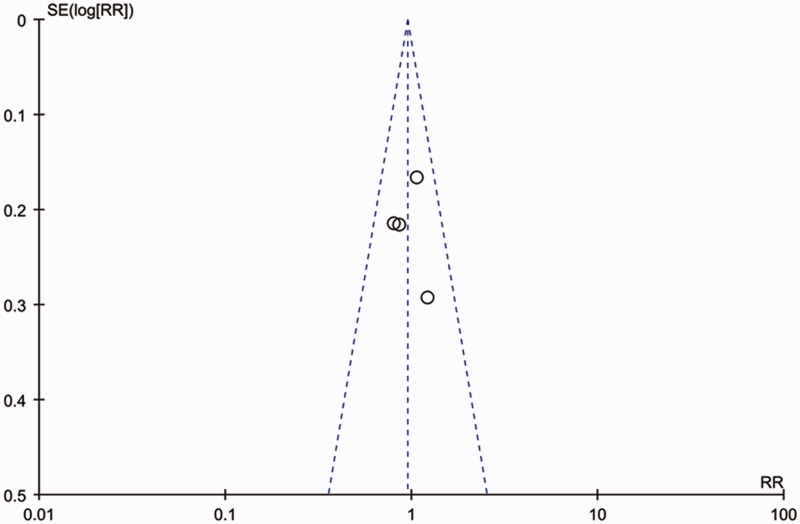
Funnel plot of trials comparing SBE and DBE. DBE = double-balloon enteroscopy; SBE = single-balloon enteroscopy.

A funnel plot analysis was conducted. The graphical funnel plot of the four studies appeared to be symmetrical, which means that publication bias is unlikely in this meta-analysis (Figure 6).

## Discussion

This study is the first meta-analysis performed to assess the efficacy and safety of single-balloon enteroscopy as compared with double-balloon enteroscopy. By summarizing the current best evidence from prospective, randomized, controlled trials, this analysis revealed that, in terms of complete small bowel visualization, there are statistically significant benefits from double-balloon enteroscopy when compared with single-balloon enteroscopy; however, DBE and SBE were similar with regard to diagnostic yield, therapeutic yield and complication rate.

Our study showed that DBE had a higher total enteroscopy rate than SBE. This is probably related to the technical differences between DBE and SBE. The balloon on the endoscope tip in DBE provides improved grip on the small bowel compared with the angled tip or suction used in SBE, and is likely to provide a longer insertion depth and therefore higher total enteroscopy rate. Insertion depth itself is only an estimation using the published technique for DBE [4], and is even more difficult to estimate the insertion depth in SBE because of the technical difference. An additional consideration is the shorter experience with SBE than with DBE, as it is a newer technique for deep enteroscopy. In fact, Takano *et al.* mention in their study that they had more experience with DBE than with SBE [12]. When they started their study, they had performed 248 DBEs and 10 SBEs. Similarly, in the study by May *et al.,* all procedures were performed by investigators experienced in DBE, each of whom had previously conducted at least 50 DBE procedures [11]; however, training in the SBE technique had been provided for only 2 months beforehand. Even in the study by Domagk *et al.,* where the endoscopists were more experienced in the SBE technique, and each trial center had performed over 50 SBE procedures before commencement of the study, they acknowledged that the endoscopists were more experienced in DBE [9]. As the technique for SBE is both newer and quite different from that of DBE, some difference may be due to the ‘learning curve’ of SBE, which might be comparable to the earlier reported learning curve of the DBE technique.

DBE and SBE showed no difference in diagnostic and therapeutic yield in this meta-analysis. This could be explained if the majority of the pathological findings were in the proximal small bowel, such that the anal approach would not add to the diagnostic rate. While the specific findings were described in each included study, the locations of these findings were not provided, so we are unable to assess whether this explains the similar diagnostic yields of DBE and SBE. Whether total enteroscopy rates have clinical impact remains controversial, because the majority of the pathological findings are usually located in the proximal small bowel, but the referred studies have not provide the precise locations of the findings and, without this data, we cannot know the precise diagnostic or therapeutic yield.

The most serious complications reported in the literature are perforations, bleeding, and acute pancreatitis [13–22]. In the combined data during our meta-analysis, we found only three complications in each of the SBE and DBE groups, i.e. 3/133 and 3/135, respectively. These complications were minor, in that the DBE group had one patient with Mallory-Weiss syndrome, one patient with post-procedure abdominal pain, and one patient with transient intra-procedure desaturation, while the SBE group had one patient with hyperamylasemia and two patients with post-procedure abdominal pain. No severe complications—including perforation, bleeding, or pancreatitis—were reported in any of these studies for either technique. While these complication rates are lower than those reported in other studies, they do suggest that deep enteroscopy using either SBE or DBE is a safe procedure.

A recent systematic review of various enteroscopy techniques, published by Lenz *et al.,* showed comparable performance (investigation time, oral intubation depth) and diagnostic yield in both enteroscopy systems [23]. These results are similar to what our meta-analysis has shown.

A recently published retrospective analysis done by Lenz *et al.* compared SBE and DBE over a period of seven years [24]. Just like our results, they also found that both these enteroscopy techniques are safe; however, their results showed a higher diagnostic yield in SBE than in DBE, which was statistically significant. They also found that SBE achieved shorter insertion depths than DBE. This is in contrast to our results, which showed similar efficacy in diagnostic yield and comparable insertion depths using both techniques. As authors pointed out, the reason for higher diagnostic yield from SBE may be due to the more focused selection of patients scheduled for small bowel diagnostics in recent years. The major limitation of this study is that it is a single-center retrospective analysis. Hence, such results should be treated with caution.

One of the four studies used carbon dioxide (CO_2_) as the insufflation gas during enteroscopy, while the other two studies used air insufflation [9]. In a previous study, Domagk *et al.* showed that using CO_2_ as the insufflation gas during enteroscopy enabled significantly extended intubation depth during double-balloon enteroscopy [25]. Meanwhile, the effect of CO_2_ on insertion depth has not been studied in SBE. Interestingly, however, the completed enteroscopy rate during both SBE and DBE was lower in the study by Domagk *et al.* than in the study by May *et al.* [11], but higher than in the study by Takano *et al.* [12]. Further studies are needed to compare the effects of CO_2_ on the enteroscopy completion rate between SBE and DBE.

Three of the four studies used the DBE system designed by Fujinon and the SBE system designed by Olympus; however, the study from May *et al.* used a modification of the Fujinon DBE system with one balloon as a proxy for the SBE procedure [11]. Certainly it needs to be born in mind that the differences in scope type (Olympus SBE system and Fujinon system) may influence the insertion depth and the rates of completed enteroscopy achieved, due to differences in technical and material composition. Future studies would be needed to examine this effect as well.

One of the limitations of these findings is that the method for insertion depth estimation with using different dedicated instruments is controversial because some authors used the published technique for DBE in SBE exploration. Additionally, different methods of enteroscopic distance estimation are used in SBE and this might have an impact on the results. Perhaps, in the exploration of the human small bowel, only the visualization of a tattoo by the opposite direction or visualization of the cecum by the oral route are the only available methods for the confirmation of a completed small-bowel enteroscopy. With only the estimated measurement of insertion in cm (in the oral introduction or withdrawal of the instrument) it is very difficult to obtain verifiable data in groups with insufficient experience of deep bowel enteroscopy. Another limitation of our study is that we could not study the effect of CO_2_ on insertion depth in SBE. Because of these limitations and small number of studies included in meta-analysis, our conclusions, despite being important and relevant additions to the current literature on deep enteroscopy, are not definitive.

The overall quality of the evidence, as assessed by the GRADE approach, was moderate for the diagnostic yield and total enteroscopies achieved. This indicates that further research is very likely to have an important impact on our confidence in the estimate of effect and is likely to change that estimate. The quality of the evidence for complication rate and therapeutic yield was judged to be very low and low, indicating low confidence in this effect estimate (Table 3). This review has some other limitations. First, this meta-analysis is a study-level—but not an individual patient-level—meta-analysis. Study-level meta-analysis can lead to biased assessments and the use of aggregated summary values brings some limitations in explaining heterogeneity. We also originally planned to analyse the differences in the preparation and examination times of SBE and DBE, assessment of the procedures by patients, and assessment of the procedures by physicians; however, due to the limited number of studies that reported relevant outcomes, and the different methods of reporting these outcomes, we did not find it appropriate to combine these in a meta-analysis. A simple analysis of funnel plots provides a useful test for the probable presence of bias in a meta-analysis, but the capacity to detect bias is limited when a meta-analysis is based on a limited number of small trials, as is the case in our review. Therefore, the results from such a meta-analysis should be treated with caution.

**Table 3.** Double-balloon *vs*. single-balloon enteroscopy for the investigation and management of small-bowel conditions

Outcomes	Illustrative comparative risks^a^ (95% CI)		Relative effect (95% CI)	No. of participants (studies)	Quality of the evidence (GRADE)
	Assumed risk	Corresponding risk			
	Control	Double-balloon *vs.* single-balloon enteroscopy			
Total enteroscopy achieved	Study population		RR = 0.37	264	⊕⊕⊕⊖
	396 per 1000	146 per 1000 (75–289)	(0.19–0.73)	(4 studies)	moderate^1^^,^^2^^,^^3^^,^^4^
	Moderate				
	378 per 1000	140 per 1000 (72–276)			
Diagnostic yield	Study population		RR = 0.95	387	⊕⊕⊕⊖
	493 per 1000	468 per 1000 (379–576)	(0.77–1.17)	(4 studies)	moderate^1^^,^^2^^,^^3^^,^^4^
	Moderate				
	510 per 1000	485 per 1000 (393–597)			
Therapeutic yield	Study population		RR = 0.78	387	⊕⊕⊖⊖
	328 per 1000	256 per 1000 (194–341)	(0.59–1.04)	(4 studies)	low^1^^,^^2^^,^^4^^,^^5^
	Moderate				
	304 per 1000	237 per 1000 (179–316)			
Complications	Study population		RR = 1.08	387	⊕⊖⊖⊖
	20 per 1000	21 per 1000 (6–84)	(0.28–4.22)	(4 studies)	very low^1^^,^^2^^,^^4^^,^^6^
	Moderate				
	28 per 1000	30 per 1000 (8–118)			

^a^The basis for the assumed risk (e.g. the median control group risk across studies) is provided in footnotes.

The corresponding risk (and its 95% confidence interval) is based on the assumed risk in the comparison group and the relative effect of the intervention (and its 95% CI).

Patient or population: patients for the investigation and management of small-bowel conditions.

CI = confidence interval; RR = risk ratio

GRADE Working Group grades of evidence:

High quality = further research is very unlikely to change our confidence in the estimate of effect.

Moderate quality: further research is likely to have an important impact on our confidence in the estimate of effect and may change the estimate.

Low quality: further research is very likely to have an important impact on our confidence in the estimate of effect and is likely to change the estimate.

Very low quality: we are very uncertain about the estimate.

^1^Serious: all studies were single-blinded and allocation concealment was done only in two studies; analysis was by intention to treat; no overt reporting biases; no conflicts of interest.

^2^No serious indirectness: this trial included only adults. This is a validated, clinically relevant outcome measure. Not downgraded.

^3^No imprecision: there are lots of events and narrow confidence intervals around the estimate of effect.

^4^Unlikely, as the search was comprehensive.

^5^Serious imprecision: there are relatively few events and wide confidence intervals around the estimate of effect.

^6^Very serious imprecision: there are relatively few events and wide confidence intervals around the estimate of effect.

## Conclusion

This meta-analysis showed that double-balloon enteroscopy and single-balloon enteroscopy are comparable with regard to diagnostic yield, therapeutic yield and complication rate but the rate of completed enteroscopy was higher with DBE than with SBE. Whether total enteroscopy rates have any clinical impact remains controversial. Based on the results of our study, these two techniques are comparable and can be used interchangeably, depending upon the expertise available at the treatment center. However, the results should be treated with caution in the light of low-to-moderate quality of evidence. There is a need for well-designed RCTs of large sample size to compare these techniques.

## References

[1] Yamamoto H Sekine Y Sato Y Total enteroscopy with a non-surgical steerable double-balloon method. Gastrointest Endosc 2001;53:216–20. 11174299 10.1067/mge.2001.112181

[2] Ell C May A Nachbar L Push-and-pull enteroscopy in the small bowel using the double-balloon technique: results of a prospective European multicenter study. Endoscopy 2005;37:613–16. 16010603 10.1055/s-2005-870126

[3] Maaser C Schmedt A Bokemeyer M Long-term efficacy and safety of double balloon enteroscopy: prospective and retrospective data from a single-center study. Scand J Gastroenterol 2010;45:992–9. 20230304 10.3109/00365521003710182

[4] May A Nachbar L Ell C Double-balloon enteroscopy (push-and-pull enteroscopy) of the small bowel: feasibility and diagnostic and therapeutic yield in patients with suspected small bowel disease. Gastrointest Endosc 2005;62:62–70. 15990821 10.1016/s0016-5107(05)01586-5

[5] Hartmann D Eickhoff A Tamm R Balloon-assisted enteroscopy using a single-balloon technique. Endoscopy 2007;39 Suppl 1:E276. 17957636 10.1055/s-2007-966616

[6] Tsujikawa T Saitoh Y Andoh A Novel single-balloon enteroscopy for diagnosis and treatment of the small intestine: preliminary experiences. Endoscopy 2008;40:11–15. 18058613 10.1055/s-2007-966976

[7] Jadad AR Moore RA Carroll D Assessing the quality of reports of randomized clinical trials: is blinding necessary? Control Clin Trials 1996;17:1–12. 8721797 10.1016/0197-2456(95)00134-4

[8] Higgins J Green S Cochrane Handbook for Systematic Reviews of Interventions. Version 5.1.0 [updated March 2011]. The Cochrane Collaboration, 2011.

[9] Domagk D Mensink P Aktas H Single- vs. double-balloon enteroscopy in small-bowel diagnostics: a randomized multicenter trial. Endoscopy 2011;43:472–6. 21384320 10.1055/s-0030-1256247

[10] Efthymiou M Desmond PV Brown G SINGLE-01: a randomized, controlled trial comparing the efficacy and depth of insertion of single- and double-balloon enteroscopy by using a novel method to determine insertion depth. Gastrointest Endosc 2012;76:972–80. 22980289 10.1016/j.gie.2012.06.033

[11] May A Farber M Aschmoneit I Prospective multicenter trial comparing push-and-pull enteroscopy with the single- and double-balloon techniques in patients with small-bowel disorders. Am J Gastroenterol 2010;105:575–81. 20051942 10.1038/ajg.2009.712

[12] Takano N Yamada A Watabe H Single-balloon *vs.* double-balloon endoscopy for achieving total enteroscopy: a randomized, controlled trial. Gastrointest Endosc 2011;73:734–9. 21272875 10.1016/j.gie.2010.10.047

[13] Kawamura T Yasuda K Tanaka K Clinical evaluation of a newly developed single-balloon enteroscope. Gastrointest Endosc 2008;68:1112–16. 18599052 10.1016/j.gie.2008.03.1063

[14] Ramchandani M Reddy DN Gupta R Diagnostic yield and therapeutic impact of single-balloon enteroscopy: series of 106 cases. J Gastroenterol Hepatol 2009;24:1631–8. 19686408 10.1111/j.1440-1746.2009.05936.x

[15] Tominaga K Iida T Nakamura Y Small intestinal perforation of endoscopically unrecognized lesions during peroral single-balloon enteroscopy. Endoscopy 2008;40 Suppl 2:E213–14. 18819062 10.1055/s-2008-1077405

[16] Aktas H Mensink PB Haringsma J Low incidence of hyperamylasemia after proximal double-balloon enteroscopy: has the insertion technique improved? Endoscopy 2009;41:670–3. 19670133 10.1055/s-0029-1214976

[17] Groenen MJ Moreels TG Orlent H Acute pancreatitis after double-balloon enteroscopy: an old pathogenetic theory revisited as a result of using a new endoscopic tool. Endoscopy 2006;38:82–5. 16429360 10.1055/s-2005-921179

[18] Heine GD Hadithi M Groenen MJ Double-balloon enteroscopy: indications, diagnostic yield, and complications in a series of 275 patients with suspected small-bowel disease. Endoscopy 2006,38:42–8. 16429354 10.1055/s-2005-921188

[19] Honda K Itaba S Mizutani T An increase in the serum amylase level in patients after peroral double-balloon enteroscopy: an association with the development of pancreatitis. Endoscopy 2006;38:1040–3. 17058172 10.1055/s-2006-944831

[20] Honda K Mizutani T Nakamura K Acute pancreatitis associated with peroral double-balloon enteroscopy: a case report. World J Gastroenterol 2006;12:1802–4. 16586559 10.3748/wjg.v12.i11.1802 PMC4124365

[21] Kopacova M Rejchrt S Tacheci I Hyperamylasemia of uncertain significance associated with oral double-balloon enteroscopy. Gastrointest Endosc 2007;66:1133–8. 17892875 10.1016/j.gie.2007.03.1085

[22] Lo SK Technical matters in double balloon enteroscopy. Gastrointest Endosc 2007;66(3 Suppl):S15–S18. 17709021 10.1016/j.gie.2007.05.046

[23] Lenz P Domagk D Double- vs. single-balloon vs. spiral enteroscopy. Best Pract Res Clin Gastroenterol 2012;26:303–13. 22704572 10.1016/j.bpg.2012.01.021

[24] Lenz P Roggel M Domagk D Double- vs. single-balloon enteroscopy: single center experience with emphasis on procedural performance. Int J Colorectal Dis 2013;28:1239–46. 23503664 10.1007/s00384-013-1673-1

[25] Domagk D Bretthauer M Lenz P Carbon dioxide insufflation improves intubation depth in double-balloon enteroscopy: a randomized, controlled, double-blind trial. Endoscopy 2007;39:1064–7. 18072057 10.1055/s-2007-966990

